# Mews Anesthesia Team: a project for in-hospital patient safety

**DOI:** 10.1186/cc9895

**Published:** 2011-03-11

**Authors:** R Oggioni, L Tadini Buoninsegni, G Iannello, C Rosati

**Affiliations:** 1 Azienda Sanitaria Firenze, Borgo San Lorenzo, Italy

## Introduction

In past years, to improve in-hospital patient safety, rapid response teams were put in place to treat, on ward's call, the patients at risk in order of their severity.

## Methods

In the Azienda Sanitaria Firenze, a dedicated group of intensivists and nurses developed a project called the Mews Anesthesia Team (MAT) in order to prevent and manage clinical deterioration of patients. This would be achieved through the activation of a rapid response team lead by an intensivist acting in accordance with an intervention flowchart. The project was based on the Modified Early Warning Score (MEWS), a validated warning score designed to alert ward nurses to patients at risk (MEWS >3) and/or to trigger MAT intervention (MEWS >5). Previously the group realized a pilot phase involving 420 patients, preceded by a 1-day dedicated course addressed to ward staff; the preliminary results showed in 12% of patients with MEWS >3, that sicker patients were located more in medical wards, while more calls/interventions of MAT were performed in surgical wards. Subsequently MEWS was plugged into the nurse electronic health record (EHR) allowing one to display automatically the score and to stratify patients according to level of care and frequency of monitoring required. The final phase of the project started from our hospital, preceded by courses for the ward staff (mostly nurses).

## Results

The performance indicator, that is nurse's compliance in MEWS, was high in surgical wards compared with medical ones (88% vs. 71%); this difference is because until recently medicine nurses fill in MEWS only by sheet records. The adherence to the interventional flowchart was extremely low in medicine wards versus surgical ones (25% vs. 60%) despite recurrent meetings with ward staff, owing to reluctance of physicians to call the MAT. There were more critically ill patients in surgery, notably in orthopedics, than in medicine wards (MEWS >3: 22% vs. 7%). As a result of MAT intervention, 30% of patients were admitted to the ICU (mostly from surgery).

## Conclusions

MAT is ongoing with good acceptance from nurses and good compliance, especially in surgery wards; the inclusion of MEWS into the EHR turned out to be a great support for the nurses. MAT represents a safety system for in-hospital patients at risk, as advocated also by the Tuscany Health Agency for Quality that, in the aim of developing good practices for patient safety, recommends MEWS for tracking and managing critical in-hospital patients.

